# 3D printing of surgical staples

**DOI:** 10.1557/s43580-022-00287-2

**Published:** 2022-05-20

**Authors:** Osama Al-Takhayneh, Holly Warren, Marc in het Panhuis

**Affiliations:** 1 School of Chemistry and Molecular Bioscience, University of Wollongong, Wollongong, NSW 2522 Australia; 2 ARC Centre of Excellence for Electromaterials Science, AIIM Facility, University of Wollongong, Wollongong, NSW 2522 Australia

## Abstract

In this work, CAD design and additive manufacturing (3D printing) are used to fabricate surgical staples. The staples were analysed on their mechanical robustness according to ASTM standard F564-17 which involved the in-house design, prototyping and fabrication (using 3D printing) of specialized grips and extension blocks. Our results indicated that staples 3D printed using carbon fibre reinforced nylon 6 (CF-PA6) exhibited a strength value of 37 ± 3 MPa coupled with an implantation-suitable ductility value of 26 ± 4%. The mechanical robustness of CF-PA6 staples subjected to immersion in simulated body fluid resulted in a reduction in stiffness and strength of 40% and 70% over 5 weeks, respectively. The carbon fibre nylon composite staples were able to handle a load of 15 kg and 5 kg prior and following immersion in simulated body fluid, respectively.

**Graphical abstract:**

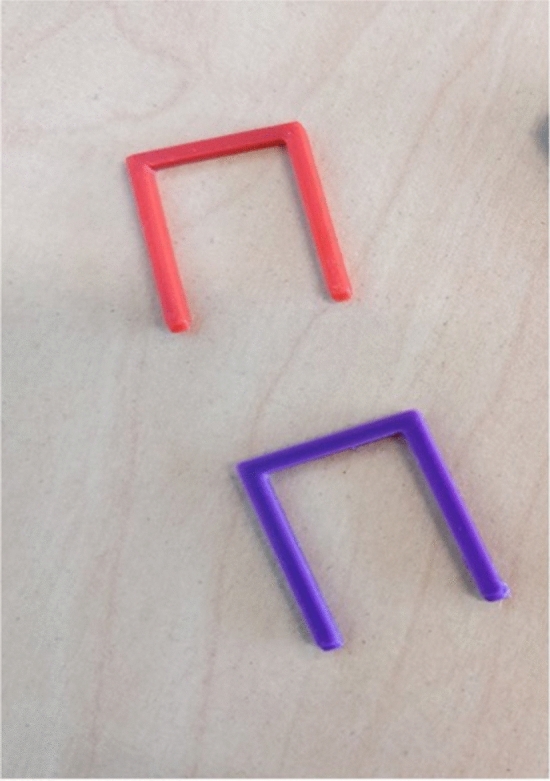

## Introduction

Injuries of bones are considered to be critical emergencies that should be handled at early stages to prevent any possibility of lasting loss of function. They include (but not limited to) fractures, cancer, and infections. In 2019, 24.5 million orthopaedic surgeries were performed worldwide [1]. It is one of the most rapidly growing surgical procedure categories [1].

The basic goal of fracture fixation is to enable fast healing of the injured bone by stabilizing it, and to return early mobility and full function of the injured extremity. Internal fixation open reduction (IFOR) is used to solve bone fractures, enable early mobilization and to overcome the limitations encountered when the treatment involves cast immobilization or skeletal traction. Most internal fixation devices rely on special devices called implants. [2, 3]

Implants are mostly made from either titanium alloy or stainless-steel composites. The abundant implants used in internal fixation surgeries can be roughly divided into few categories: plates, pins, screws, wires, rods/nails, staples, and clamps. The main three materials used in implants fabrication are metals, ceramics, and polymers. Drawbacks of metals include mechanical mismatch (e.g. strength, elastic modulus and toughness) with human bones tissues, which results in a stress shielding effect leading to prosthetic loosening, osteolysis, and periprosthetic fracture [4]. In addition, long-term implantation of metals can trigger hypersensitivity reaction and initiate osteolysis [5]. Ceramics used in implants include mainly calcium phosphate, aluminium oxide, zirconia, hydroxyapatite, and glass ceramics. These materials provide very high compression resistance which is very useful specially in dental applications [6]. However, their mechanical toughness, ductility, and brittleness make them unsuitable for load bearing cases [7]. As a result of these drawbacks (in metals and ceramics), polymeric materials are being investigated as suitable materials for internal fixation devices.

Traditional techniques for producing implants include casting and/or moulding. Other techniques involving additive manufacturing (3D printing) as a rapid prototyping tool are gaining popularity [8]. Rapid prototyping (RP) is a set of techniques used to fabricate highly accurate parts out of CAD (Computer Aided Design) models [9]. Additive manufacturing combined with RP techniques have been employed to provide solutions in dentistry [10], bone tissue engineering [11], orthopaedics [12], organ/tissue printing [13], and medical equipment during COVID-19 pandemic [14]. 3D printing through fused deposition modelling (FDM, sometimes referred to fused filament fabrication) is one of the most widely used types of rapid prototyping techniques. FDM printers work by extruding polymer-based filaments through a heated nozzle, by melting the filament the prototype is formed layer by layer until its complete [15].

In this study, we describe the in-house design, prototyping and fabrication (using 3D printing) of specialized grips and extension blocks required for the characterization of 3D printed (through fused deposition modelling) surgical staples using mechanical analysis and immersion in simulated body fluid (SBF).

## Materials and methods

### Materials

The filaments used for 3D printing were poly(lactic acid) (PLA), poly(ethylene terephthalate) (PETG), carbon fibre reinforced poly(ethylene terephthalate) (CF-PETG), carbon fibre reinforced nylon 6 (CF-PA6) and carbon fibre reinforced nylon 12 (CF-PA12), all obtained from Cubic Technologies (Australia). A commercially available bone fixation staple was obtained from Smith and Nephew (Australia). All salts required for simulated body fluid were obtained from Sigma-Aldrich (Australia).

### Design and 3D printing of staples

**Fig. 1:**
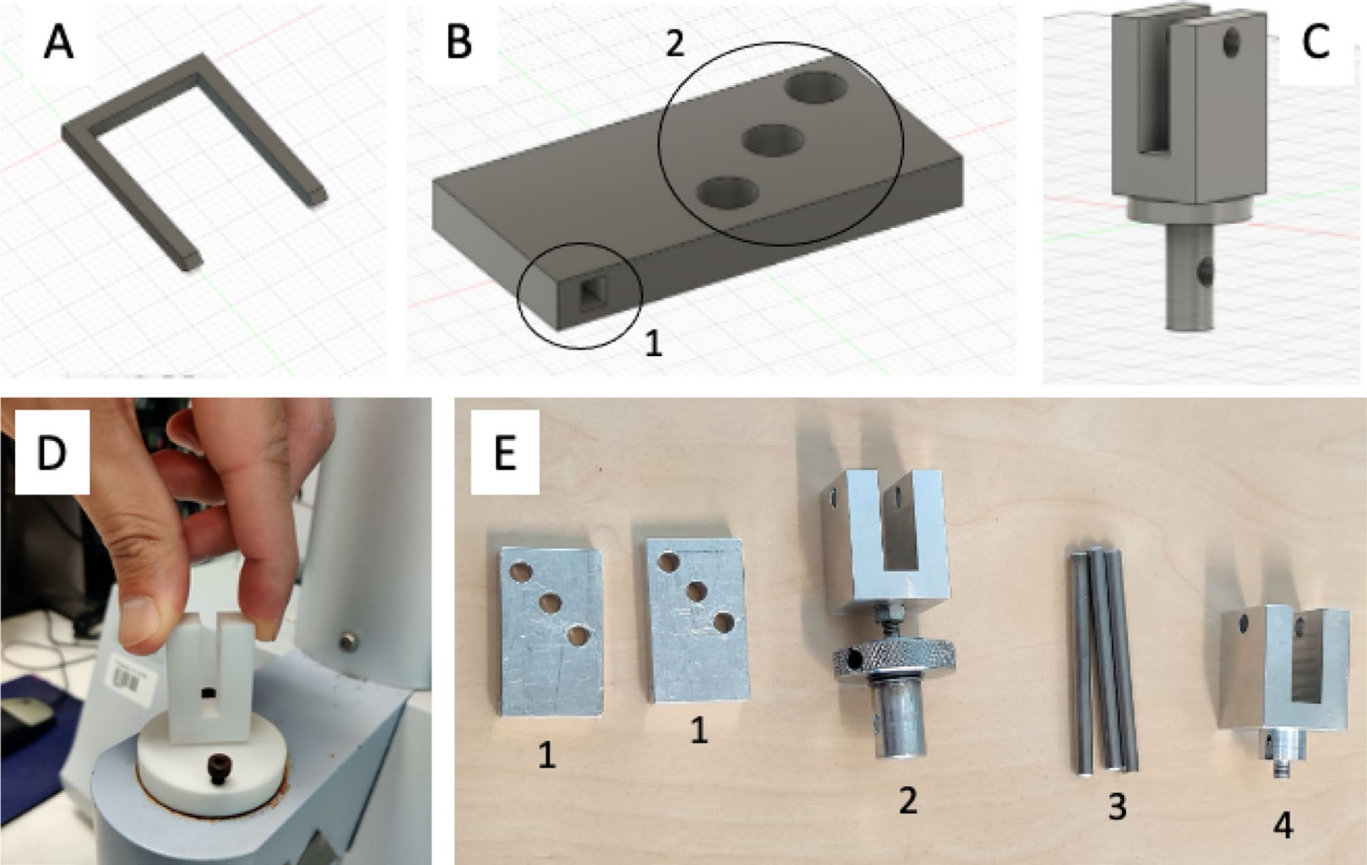
**A** CAD image of staple with dimensions length (23.2 mm), width (14.4 mm) and thickness (2 mm). **B** CAD image of extension block with length (25 mm), width (12.5 mm) and thickness (5 mm). Numbers 1 and 2 indicate staple leg position and different load positions, respectively. **C** CAD design of the bottom grip that connect to the bottom part of the mechanical analyser. **D** 3D printed version (in PLA) of the CAD design shown in **C**) connected to the mechanical analyser. **E** Extension blocks, and grips fabricated in aluminium. Number 1–4 indicate extension blocks to hold the staple from both sides, a bottom grip that connects to bottom part of the mechanical analyser, stabilizing pins to hold the extension blocks and the grips together, and upper grip that connects to the load cell of the mechanical analyser, respectively

A standard bone staple called Richard staple or Richard U-staple [8] was designed (from scratch) using CAD (Autodesk Fusion 360, Fig. 1A). The main dimension of the staples are length (23.2 mm), width (14.4 mm), and thickness (2 mm). All 3D printing was carried out using an in-house modified FDM printing (Creality 3D CR-10S Pro v2, with changes to nozzle, build plate, tubing, and hotend) with 100% infill.

### Thermogravimetric analysis

Samples parts (10–20 mg) of 3D printed staples were analysed using thermogravimetric analysis (Netzsch, TG 209 F1) with crucibles (alumina ceramic, Shimadzu). The heat rate was set to increase 5 °C/min under ambient air from 20 to 950 °C.

### Mechanical testing

A sample holder was designed (using CAD, Fig. 1B–C), rapid prototyped (in PLA, Fig. 1D), and fabricated (in aluminium, Fig. 1E) in-house following the specifications provided in the ASTM Standard F564-17 for validating commercial surgical staples [16]. The method designates the design of a sample holder consisting of two specialized metal extension blocks for proper placement of the staple during the test (Fig. 1B–E).

**Fig. 2:**
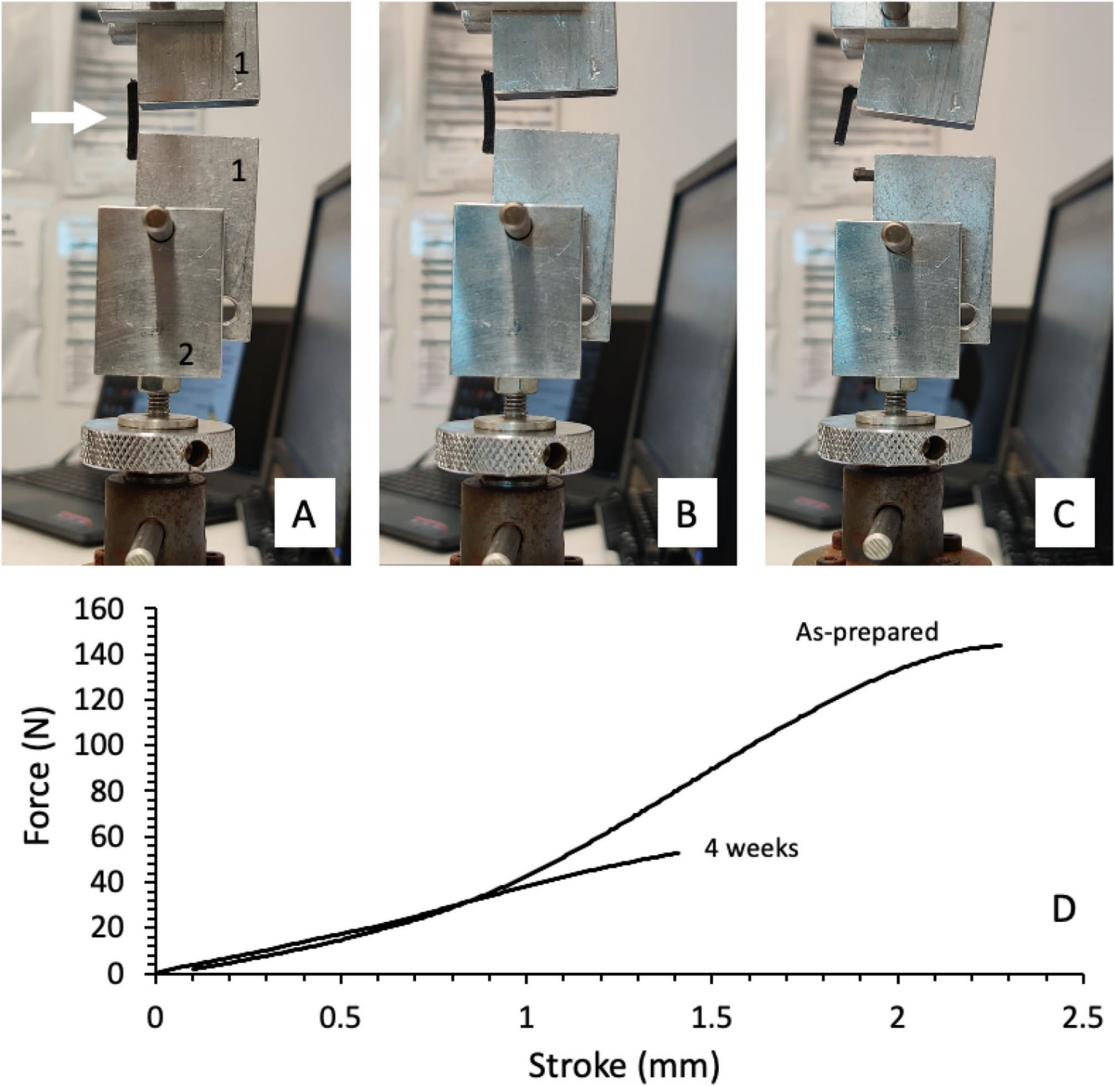
**A** Image showing staple loaded in the sample holder consisting of extension blocks and grips. Arrow points to staple. Number 1 and 2 indicate extensions block and bottom grip connected to mechanical analyser. **B** and **C** images showing staple under tension and after failure, respectively. **D** Comparison of the force as a function of stroke for 3D printed staples printed in carbon fibre reinforced nylon 6 and following immersion in simulated body fluid for 4 weeks

During testing, the legs of each staple are fitted into fixation holes in each extension block with minimal clearance to restrict bending of the staple within the hole (Fig. 2A). The extension blocks were connected to the metallic grips and connected to a universal mechanical analyser (EZ-S, Shimadzu, 500 N load cell). All tests were carried out at 21 °C at a rate of 1 mm/min. Stress, strain, modulus, strength and ductility were evaluated from force divided by cross-sectional area, stroke divided by distance between grips, slope of stress–strain curve, stress at failure and strain at failure, respectively.

Failure was identified in a complete separation of staple component, a visible crack, or a significant drop in applied force. Video analysis was used to assess for signs of slippage or micro-cracks, which resulted in rejection of the data.

### Simulated body fluid

Simulated body fluid (SBF) was prepared by adding quantities of sodium chloride (NaCl, 7.996 g), bicarbonate of soda (NaHCO_3_, 0.350 g), potassium chloride (KCl, 0.224 g), dipotassium phosphate (K_2_HPO_4_⋅3H_2_O, 0.228 g), magnesium chloride (MgCl_2_, 0.305 g), calcium chloride (CaCl_2_, 0.278 g), sodium sulphate (Na_2_SO4, 0.071 g), tris(hydroxymethyl)aminomethane [(CH_2_OH)3CNH_2_), 6.057 g], respectively, into 500 ml deionized water (resistivity 18 MO cm) at 37 ± 3 °C under magnetic stirring. The pH was adjusted to 7.4 to match the blood plasma [17] by adding HCl and deionized water to increase the total volume to 1 L. Staples were immersed in SBF inside a sterile polystyrene containers, which were kept for up to 5 weeks at 37 ± 1 °C. Sets of staples (three) were taken out at day 2 after immersion, then at every week for the duration of the test. Each set of three staples was dried using a paper towel and subjected to mechanical testing at 21 °C.

## Results and discussion

Staples were prepared using 3D printing using different polymer composite materials and subjected to mechanical testing prior and during immersion in simulated body fluid. There was excellent agreement between the dimensions of all the staples prepared using each of the filaments. The average dimensions of the 3D printed staples were 14.4 ± 0.2 mm (width), 23.4 ± 0.2 mm (length), 2.1 ± 0.1 mm (thickness) and weight (0.29 ± 0.01). Thermogravimetric analysis (TGA) showed that the composite materials contained about 20% (by weight) carbon fibre (data not shown). The moisture content was estimated below 2%.

### Mechanical characteristics of as-prepared staples

The mechanical characteristics of the 3D printed staples were tested until failure (Fig. 2). A summary of the mechanical characteristics of the as-prepared staples is presented in Table 1. Our data were benchmarked against a commercially available aluminium alloy staple.

**Table 1:** Mechanical properties of 3D printed staples

Staples	Modulus (MPa)	Strength (MPa)	Ductility (%)
Al Alloy	210 ± 20	120 ± 10	18 ± 2
PETG	50 ± 10	13 ± 4	21 ± 2
CF-PETG	150 ± 10	20 ± 1	13 ± 1
CF-PA12	100 ± 20	28 ± 2	48 ± 2
CF-PA6	150 ± 20	37 ± 3	26 ± 4

All mechanical testing performed at 21 °C. PETG, CF-PETG, CF-PA6 and CF-PA12 indicate poly(ethylene terephthalate), carbon fibre reinforced poly(ethylene terephthalate), carbon fibre reinforced nylon 6 and carbon fibre reinforced nylon 12, respectively. Al Alloy refers to a commercial sample of similar dimensions to the design used for 3D printed staples. Strength and ductility values for Al alloy staple are as per the limits of our load cell (500 N), i.e. not failure. All reported values are mean calculated with 95% confidence interval (*n* = 10)

It is well known that carbon fibre leads to reinforcement of the polymer matrix but at a cost of ductility. We demonstrated this effect, through a comparison between staples printing using PETG filaments with and within carbon fibre. Our testing confirmed that the CF-PETG filament resulted in an increase in strength (from 13 ± 4 to 20 ± 1 MPa), but a decrease in ductility (from 21 ± 2 to 13 ± 1%).

Carbon fibre reinforced nylon 6 exhibited the highest tensile strength (37 ± 3 MPa) followed by CF-PA12 (28 ± 2 MPa) and CF-PETG (20 ± 2 MPa). As expected, these strength values are lower than the strength exhibited (125 MPa) of the commercially available staple. Modulus values of CF-PETG and CF-PA6 were approximately similar (150 MPa) and larger than the values exhibited by CF-PA12 (104 ± 15) and in the same order of magnitude as the commercially available staple. The ductility of the staples varied between the different materials with CF-PA12 exhibiting the highest ductility value of 48 ± 2%. This value exceeds the recommended ductility range (25–30%) for surgical staples [18]. In contrast, the ductility of CF-PETG is too low, while the ductility of CF-PA6 (26 ± 4%) is within the recommended range. Based on these results, staple 3D printed in CF-PA6 were used for analysis of the impact on mechanical characteristics following immersion in simulated body fluid.

### Immersion in simulated body fluid

The staple’s dimensions, weight, and mechanical robustness were measured at regular intervals during the immersion in simulated body fluid. No changes in the dimensions of the staples were observed, and there was a minor increase in weight (of 0.02 g) following immersion. Table 2 shows the average weights and tensile test results of CF-PA6 before and after SBF immersion. Figure 2D shows an example of typical tensile test results before and after immersion (for 4 weeks).

**Table 2:** Mechanical properties and weight of staples printed in CF-PA6 (carbon fibre nylon 6 composite) as a function of immersion in simulated body fluid

Time (days)	Modulus (MPa)	Strength (MPa)	Ductility (%)	Weight (g)
0	150 ± 20	37 ± 3	26 ± 4	0.29 ± 0.01
2	140 ± 20	31 ± 2	18 ± 2	0.29 ± 0.02
7	170 ± 10	25 ± 2	14 ± 2	0.31 ± 0.02
14	70 ± 10	11 ± 2	14 ± 1	0.32 ± 0.02
21	100 ± 10	13 ± 1	15 ± 1	0.32 ± 0.02
28	100 ± 10	12 ± 2	15 ± 1	0.32 ± 0.02
35	80 ± 10	11 ± 1	15 ± 1	0.32 ± 0.02

All mechanical testing performed at 21 °C. All reported values are mean calculated with 95% confidence interval (*n* = 10)

During SBF immersion of CF-PA6 staples, it is suggested that the composite experienced signs of hydrolysis of the molecular backbone in PA6. After one week of SBF immersion, the strength and ductility decreased to 25 ± 2 MPa and 14 ± 2%, respectively. The decrease continues until week 2 immersion after which stiffness (modulus) and strength reach plateau values of 90 ± 10 MPa and 12 ± 1 MPa, respectively. This corresponds to a reduction of 40% in stiffness and 70% in strength.

It is likely that the decrease in mechanical robustness can be attributed to hydrolysis as previously reported for PA6 [19, 20]. It has been suggested that this results in a extensibility of the macromolecular chains. In addition, the presence of salts in the SBF may also play a role [21].

For practical reasons, it is worthwhile to convert the strength values of the 3D printed staples from stress into load. Prior to immersion in SBF, staples printed in CF-PA6 were able to handle a stress of 37 MPa which is equivalent to a load of 150 N (or 15 kg) at failure (see Fig. 2D). The plateau value for strength (12 MPa) reached after 2 weeks of immersion translates to the staples being able to withstand a load of 5 kg (or 50 N).

Here, we suggest that polymeric staples are easy to shape, design, and manufacture, which gives surgeons the flexibility of choosing the optimum design and shape for the patients need. It is well known that, unlike metal alloys, polymers can be modified to be biodegradable which could eliminate the potential need for a second surgery to remove internal implants in some cases. Future efforts in this area should investigate other types of composite materials and additive manufacturing methods (e.g. fused filament fabrication) to improve on the mechanical robustness of 3D printed staples.

## Conclusions

Surgical staples were designed using CAD and fabricated using additive manufacturing (3D printing). The mechanical characterization of the polymeric staples was achieved ASTM standard F564-17 using specialized metallic grips and extension blocks, designed using CAD, prototyped with 3D printing, and followed by fabrication in aluminium.

Carbon fibre reinforced nylon 6 (CF-PA6) exhibited the highest strength (37 ± 3 MPa) and an optimum ductility for implantation (26 ± 4%) of the 3D printed samples. Immersion of CF-PA6 staples in simulated body fluid revealed that over the course of 5 weeks the mechanical robustness is reduced, i.e. a reduction in stiffness and strength of 40% and 70%, respectively. Our data indicate that following immersion in SBF, staples printed in CF-PA6 had a mechanical robustness that could handle a load of 5 kg.

This paper contributes to the deployment of 3D printing as a useful tool for designing and rapid prototyping surgical staples. Future areas of interest could include the use of additive manufacturing techniques (such as 3D printing) and polymer composite materials to other types of implants, e.g. bone plates.

## Declarations

### Data availability

On behalf of all authors, the corresponding author states that data will be made available upon request.
